# Structural stabilities, robust half-metallicity, magnetic anisotropy, and thermoelectric performance of the pristine/Ir-doped Sr_2_CaOsO_6_: strain modulations†

**DOI:** 10.1039/d5ra02453f

**Published:** 2025-05-21

**Authors:** Samia Shahzadi, Ihab Mohamed Moussa, Sohail Mumtaz, S. Nazir

**Affiliations:** a Department of Physics, University of Sargodha 40100 Sargodha Pakistan safdar.nazir@uos.edu.pk +92 334 9719060; b Department of Botany and Microbiology, College of Science, King Saud University P.O. Box 2455 Riyadh 11451 Saudi Arabia; c Department of Chemical and Biological Engineering, Gachon University 1342 Seongnamdaero, Sujeong-gu Seongnam-si 13120 Republic of Korea sohail.ahmed2015@gmail.com

## Abstract

Half-metallic (HM) ferromagnetic (FM)/ferrimagnetic (FIM) materials with a large energy-gap (*E*_g_) and high magnetocrystalline anisotropy energy (MAE) are receiving consideration for their potential usage in solid-state electronic devices. This study explores various traits of the pristine (prs.)/Ir-doped (dop.) Sr_2_CaOsO_6_ structure using *ab initio* calculations, where Ir is doped at the Os-site. To determine the synthesis feasibility of the structures under ambient conditions, the formation energy, elastic constants, and phonon curves are determined. The prs. structure manifests a FM semiconducting nature with an *E*_g_ of 0.048 eV. Strikingly, the Ir-dop. structure becomes HM FIM because additional electrons provided by the dopant (Ir) cause a repulsion in the Os t^2^_2g_ spin-minority channel, resulting in conductivity. Conversely, an *E*_g_ of 1.15 eV in the spin-majority channel exists, which is high enough to keep the HM state stable. The computed partial spin-moment on the Os in the prs. system is 1.19 *μ*_B_. In the Ir-dop. system it is 1.09/−1.39 *μ*_B_ on the Os/Ir ion holding an Os^+6^/Ir^+4^ state with electronic distributions of 5d^2^(t^2^_2g_↑t^0^_2g_↓e^0^_g_↑e^0^_g_)/5d^5^(t^3^_2g_↑t^2^_2g_↓e^0^_g_↑e^0^_g_) with 
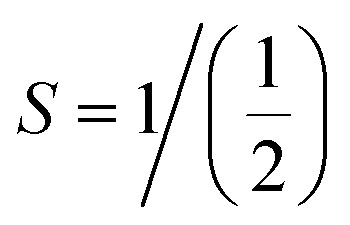
. Further, the spin-magnetization density isosurfaces assist in determining the *m*_s_ values and FM/FIM state of the prs./Ir-dop. system holding a Curie temperature (*T*_C_) of 185/171 K. Besides this, we computed the thermoelectric properties of the prs./Ir-dop. motifs; the figure of merit (0.33/0.02), Seebeck coefficient (147/30 μV K^−1^), and low thermal conductivity (0.21/0.71 × 10^19^ Ωm^−1^ s^−1^) at 300 K highlight their potential for conversion devices. Interestingly, a semiconducting-to-HM transition is predicted at a crucial compressive strain of −3% in the prs. structure. Conversely, the HM state in the dop. structure displays robustness against strain. Additionally, it is shown that an applied tensile strain can significantly improve *ZT*, while compressive strains illustrate a positive impact on the *T*_C_ value.

## Abstract 2

This study explores various traits of the pristine (prs.)/Ir-doped (dop.) Sr_2_CaOsO_6_ structure using *ab initio* calculations, where Ir is doped at the Os-site.

## Graphical abstract

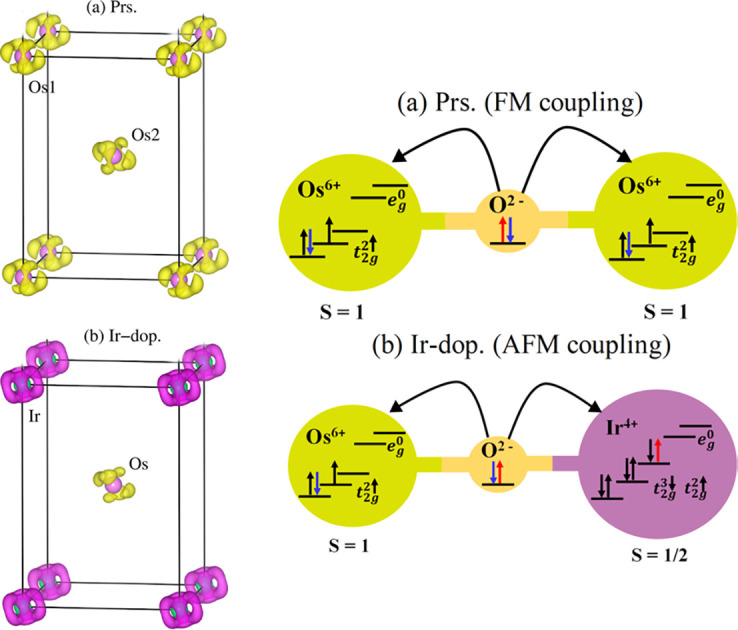

## 1 Introduction

Depending on the B and B′ cations, double perovskite oxides (DPOs) display a wide range of ferromagnetic (FM), ferrimagnetic (FIM), and anti-ferromagnetic (AFM) spin ordering (SO) in addition to half-metal (HM)^1,2^ and semiconducting (SC)/insulating phases,^3–5^ and an insulator-to-HM transition.^6^ Materials research continues to focus on the insulator-to-metal transitions (IMTs) because of the conceptual gaps that lead to an inability to regulate the electronic conduction in prospective magnetic memory devices.^7^ Likewise, structure modifications in the crystals allow the material to transform from a band insulator to Mott insulator (MI).^8^ In particular, DPOs based on the 5d transition metals (TMs) provide an ideal environment for orbital, charge, and lattice degrees of freedom. In this respect, osmium-based materials have attracted a lot of interest due to the successful synthesis process of the oxides, which generates a variety of unusual phases. For example, a MI FM state in Ba_2_NaOsO_6_ (ref. 9) and a magnetic insulating state in Sr_2_MOsO_6_ (where M = Cu/Ni)^10^ are predicted. Interestingly, the HM FIM state has been discovered with an energy gap (*E*_g_) of 0.40 eV in the spin-minority channel (N^↓^) of Sr_2_CuOsO_6_,^11^ where the spin-majority channel (N^↑^) is metallic. The combined effect of electron correlation and spin–orbit coupling (SOC) results in a FIM MI character of the Ca_2_FeOsO_6_ structure.^12^ Similarly, a half semi-metallic FIM phase in Sr_2_CrOsO_6_ is theoretically predicted within the GGA+U+SOC method^13^ owing to a FIM MI state with a massive *T*_C_ (Curie temperature (temp.)) of 725 K.^14^ Moreover, the Sr_2_CrOsO_6_ system displays a compensatory HM behavior without the influence of SOC. However, when SOC is included, a net magnetic moment of 0.54 *μ*_B_ is obtained,^13^ which exposes that the dominating influence of SOC cannot be properly attributed to the magnetic moment correction.

Systems with high *T*_C_ and HM FM/FIM stable ground states are in huge demand for the fields of spintronics,^15–17^ magnetoelectronics,^18^ magnetodielectric capacitors,^19,20^ and data storage devices.^21^ It has been discovered that enhancing the antisite disorder deficiencies and grain boundaries may improve the physical aspects of DPOs.^22,23^ Hence, the d-orbital occupancy can be effectively regulated by doping the B/B′ site with various TMs to significantly improve magnetic exchange interactions between them, which often results in high-*T*_C_ HM systems.^24,25^ The *T*_C_ for Sr_2_CrOsO_6_ jumps dramatically from 490 to 660 K when the larger Sr cation is substituted at the Ca-site.^26–28^ Furthermore, the inclusion of disordered BO_6_ and B′O_6_ octahedra accumulating irregularly leads to a crystal field splitting and enhances the unusual electronic and magnetic features.^29^ The structural distortions are reduced when Ba is substituted at the Sr-site in ALaNiOsO_6_ (A = Sr, Ba) and the Weiss temp. (*θ*_w_) changes from negative to positive.^30^ Experimental observations reveal that doping of Ni^2+^ at the Fe-site in Sr_2_Fe_1.5_Mo_0.5_O_6−*δ*_ leads to competition between Fe^3+^/Mo^5+^ and Fe^2+^/Mo^4+^ along with induced conductivity.^31^ By adopting a similar strategy, Bhandari *et al.*,^32^ utilizing density functional theory (DFT) calculations, predicted that the system enters into an HM state when Ni^2+^ is doped (dop.) at the Cr^3+^ site (electron doping) in the FIM MI Ca_2_CrOsO_6_ system. In addition to doping of various elements at the A or B site in A_2_BB'O_6_, the doping of osmium at the B′ site in Sr_2_CrReO_6_ significantly increases the *T*_C_ and causes an HM-to-insulator transition.^33^ However, partial magnetizations in Sr_2_CrReO_6_ at the Re-site dramatically decrease at a 40% W-amount and a drop in *T*_C_ is also observed with an increase in dopant (W) concentration.^34^

In the same way, the strain approach is one of the most effective means of adjusting or controlling a material’s traits by altering the lattice parameters under various growth circumstances.^35,36^ For example, when a Sr_2_FeMoO_6_ film is formed on a SrTiO_3_ substrate, lattice mismatch causes compressive (comp.) strain of −1.2%, leading to an HM FM state.^36,37^ Furthermore, at −8% comp. and +1% to +5% tensile (tens.) strains, a FM SC to metal and HM transition is verified in La_2_FeMnO_6_, respectively.^38^ Likewise, in the Lu_2_NiIrO_6_ DPO, a FIM SC to HM transition is noted under hydrostatic stress of −6% and biaxial strain of −8%.^39^ Besides this, experimental observations reveal that lattice mismatch occurs when Re_2_NiMnO_6_ thin films (where Re = La, Pr, Nd, Sm, and Y) grow on a LaAlO_3_ substrate; the B(B′)O_6_ octahedra are further deformed by induced strain, which improves the films’ magnetic qualities and serves as an effective approach to get the maximum *T*_C_ in DPOs.^40^ Interestingly, a magnetic transition from the FM SC to the HM FM state in La_2_FeMnO_6_ is demonstrated by DFT investigations with the application of a biaxial ([110]) strain ranging from 0% to +10%.^38^ Moreover, under volume compression, a HM state with an extremely high *T*_C_ of 624 K is predicted in the Ca_2_MoOsO_6_ structure.^41^

The above-mentioned considerations make it abundantly evident that B′-site doping in DPOs is also a suitable method for altering physical aspects. Thus, we theoretically study the effect of Ir-doping at the Os-site on the distinct traits of FM SC Sr_2_CaOsO_6_ (SCOO). Because of the partial filling of the 5d orbitals of the dopant (Ir) and the host (Os), a SC-to-metal transition (MT) is observed in the Ir-doped (dop.) structure. Each system shows an appropriate amplitude of magnetic anisotropy energy (MAE), which improves the system’s functionality for the data storage devices.^21^ Along with this, we do a thorough examination of the material's thermoelectric (TE) qualities for its use in renewable energy devices. Additionally, detailed investigations of the biaxial ([110]) strain effect on the physical behavior of the systems were conducted, which give interesting outputs.

## 2 Computational and structural details

A full-potential linearized augmented plane-wave approach based on spin-polarized (SP) DFT, as implemented in the WIEN2K code,^42^ was employed for the current calculations. The exchange–correlation functional utilized in this study, which is derived from the generalized gradient approximation (GGA) combined with on-site Coulomb interaction (GGA+U), is used, keeping *U* as 2.8/2.6 eV on the 5d states of the Ir/Os ion.^43^ Additionally, because of the heavy Os/Ir element, SOC effects are also employed in the scalar relativistic form. In the wave-function expansion within the atomic spheres, *ł*_max_ = 12, *R*_mt_ × *K*_max_ = 7, and *G*_max_ = 24 within the irreducible wedge of the Brillouin zone is taken. A 6 × 6 × 4 *k*-mesh with 76 points is found to be highly converged. Also, the atomic positions of the ions are fully relaxed by lowering the overall forces below 5 mRy a.u.^−1^. Self-consistency of the system is assumed for a total energy (*E*_t_)/charge convergence to 10^−5^ Ry/10^−5^ C. Moreover, Boltzmann's theory is used to compute the TE parameters using the relaxation-time approximation, as defined in the BoltzTrap code.^44^

**Fig. 1:**
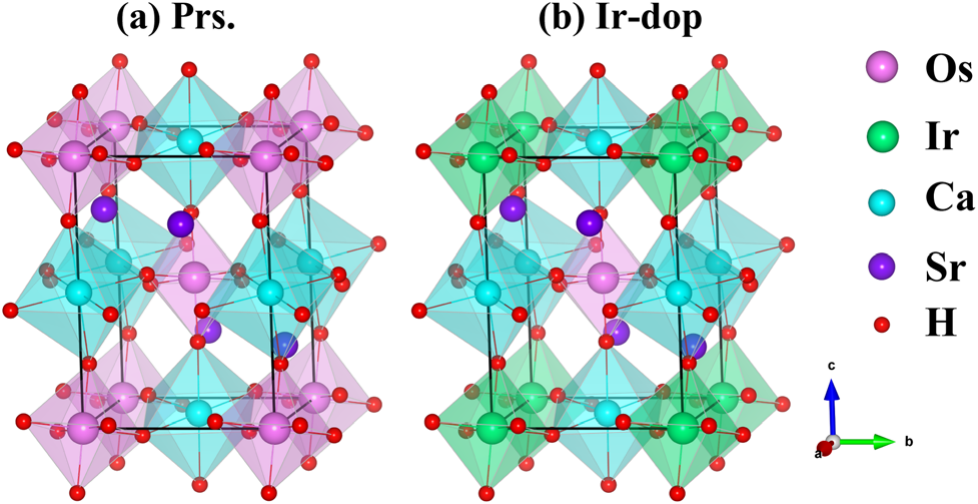
Crystal diagram of the (a) prs. and (b) Ir-dop. Sr_2_CaOsO_6_ structures.

The monoclinic SCOO keeps CaO_6_ and OsO_6_ octahedra alternately organized in a rock-salt pattern, providing a full 1 : 1 ordering with space group no. 14 (*P*2_1_/*n*). The experimental lattice constants are *a* = 5.7643, *b* = 5.8191, and *c* = 8.1796 Å with *β* = 90.22°.^45^ In the primitive unit cell of SCOO, 4/2/2/12 Sr/Ca/Os/O ions exist. The atomic coordinates for the Sr, Ca, Os, O_1_, O_2_, and O_3_ are (0.0104, 0.0334, 0.2490), (0.5, 0, 0), (0.5, 0, 0.5), (0.265, 0.312, 0.028), (−0.186, 0.234, −0.038), and (−0.088, 0.479, 0.227), correspondingly. The Ir-dop. SCOO structure is designed by replacing one of the Os ions with one Ir in its primitive unit, as reported in the previous experimental^46,47^ and theoretical^5,48^ works, where it changes the *P*2_1_/*n* to *P*1 structural symmetry. The crystal structure of the prs./Ir-dop. motif is presented in Fig. 1(a) and (b).

## 3 Results and discussion

### 3.1 Unstrained systems

**Fig. 2:**
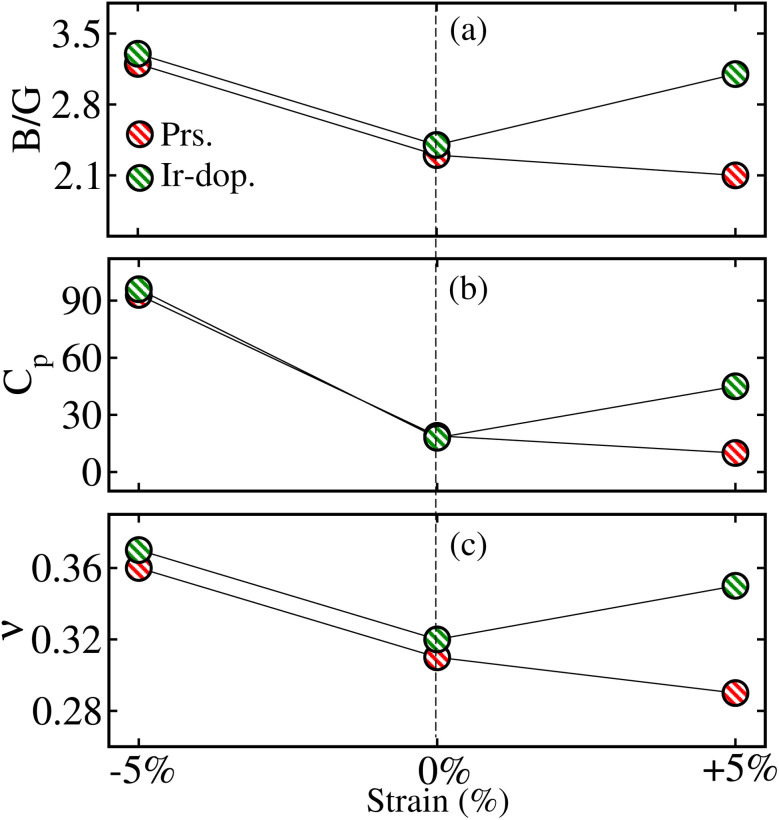
Computed (a) Pugh's ratio (*B*/*G*), (b) Cauchy's pressure (*C*_P_), and (c) Poisson’s ratio (*ν*) in the prs./Ir-dop. Sr_2_CaOsO_6_ structure for −5% (compressive)/0% (unstrained)/+5% (tensile) strain.

**Fig. 3:**
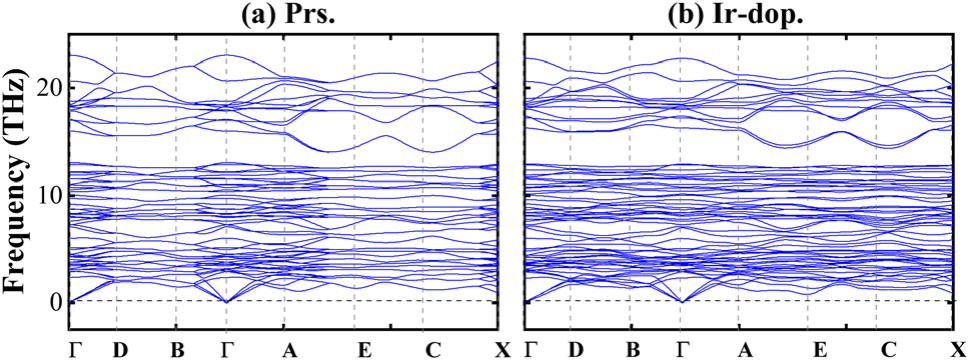
Computed phonon dispersion curves for the (a) prs. and (b) Ir-dop. Sr_2_CaOsO_6_ structures.

First, the thermodynamic stability of the structures is examined by determining the formation enthalpy (Δ*H*_f_) as follows:1
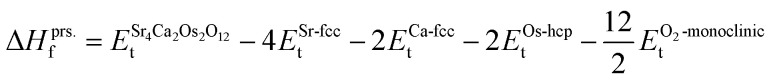
2
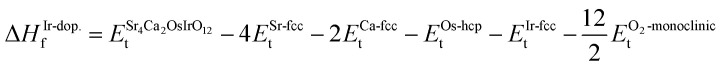

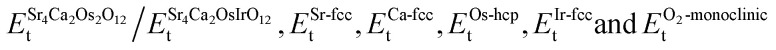
 represent the *E*_t_ of the prs./Ir-dop. SCOO, Sr (*Fm*3̄*m*-225), Ca (*Fm*3̄*m*-225), Os (*P*6_3_/*mmc*-194) and Ir (*Fm*3̄*m*-225) atoms and oxygen (*C*2/*m*-12) molecule in their respective ground states, correspondingly. To prevent the overestimation of the Δ*H*_f_ value, the suggested adjustments by Wang *et al.*,^49^ are also considered. The estimated Δ*H*_f_ of the prs./Ir-dop. structure is −29.25/−25.85 eV, where the “−” sign ensures the thermodynamic stability of the system. Next, to determine the mechanical stability of both structures, we calculated the elastic tensors (*C*_*ij*_) by generating six finite constants using conventional strain and stress relations.^50,51^ The computed 13 independent elastic stiffness tensors for the monoclinic systems are listed in Table 1, which fulfilled the basic requirements and Born stability criteria^52^ for mechanical stability. Additionally, Fig. 1S of the ESI† displays the bulk modulus (*B*), shear modulus (*G*), and Young modulus (*Y*) of both systems. *B* measures a compound's ability to deform in response to the pressure surrounding its surface, commonly known as hardness. Along with this, a higher value of *G* suggests that the material is stiffer and less likely to deform under shear forces, whereas lower values indicate that it is more flexible or ductile. *Y* is also called the modulus of elasticity and is a measure of a material's stiffness. A high value means that the materials are stiff and resistant to deformation under stress, while lower denotes a more elastic and easily deformed material. Hence, the *B*, *G*, and *Y* values of the prs./Ir-dop. system are 129/128, 145/142, and 55/54, respectively. It shows that the prs. system is stiffer than the doped one. Furthermore, we computed Pugh's ratio (*B*/*G*), Cauchy's pressure (*C*_P_), and Poisson's ratio (*ν*), which illustrate whether a compound is ductile or brittle,^53^ as displayed in Fig. 2. If the *B*/*G* value is less than 1.75, *ν* is less than 0.25, and the *C*_P_ value is less than 0, this reveals a brittle nature. In contrast, if the *B*/*G* value is greater than 1.75, *ν* is greater than 0.25, and the *C*_P_ value is greater than 0, this results in a ductile nature.^54^ Hence, our computed values of the *B*/*G* (see Fig. 2(a)), *C*_P_ (see Fig. 2(b)) and *ν* (see Fig. 2(c)) for both structures demonstrate that they are ductile. Finally, phonon calculations were performed for both structures to confirm their dynamical stability. The determined phonon spectra for the prs./Ir-dop. systems are plotted in Fig. 3(a) and (b). In principle, each atom typically contributes three phonon branches with the total number of phonon branches in a unit cell being precisely proportional to the three times number of atoms. There are 3*n* total branches, which include 3*n* − 3 optical modes and 3 acoustic modes.^55^ Both structures contain 20 atoms in their basic unit cell and have 60 vibrational modes including 3 acoustic modes along with 57 optical modes. The phonon dispersion curves do not contain any negative frequencies for both structures (see Fig. 3), indicating that they are dynamically stable as well.

**Table 1:** Computed 13 independent elastic constants (*C*_*ij*_) of the prs. and Ir-dop. Sr_2_CaOsO_6_ structures under −5%/0% (unstrained)/+5% biaxial ([110]) strain

Systems	Strain	*C*_11_	*C*_12_	*C*_13_	*C*_15_	*C*_22_	*C*_23_	*C*_25_	*C*_33_	*C*_35_	*C*_44_	*C*_46_	*C*_55_	*C*_66_
prs.	−5%	203.70	144.29	164.91	6.71	204.85	162.51	16.06	258.55	−4.48	69.14	−4.86	62.03	101.73
	0%	193.76	113.58	90.42	3.94	188.95	78.33	−3.98	222.13	4.44	59.37	−6.03	49.71	67.45
	+5%	144.22	68.45	52.47	−0.95	128.78	56.99	−2.54	183.14	0.98	46.39	−2.03	32.00	45.07
Ir-dop.	−5%	201.35	144.26	165.27	5.13	200.23	162	14.39	252.84	−6.38	66.81	−3.98	60.87	102.21
	0%	190.53	113.98	90.20	4.09	183.40	75.84	−3.81	222.68	3.36	57.46	−5.58	47.99	68.69
	+5%	189.78	94.99	76.84	7.37	149.68	96.63	14.68	187.91	−17.41	51.62	−4.55	25.27	67.67

To find the magnetic ground state (MGS) of the prs. system, we plotted the computed *E*_t_ for the non-magnetic (NM), FM, and AFM MGS spin ordering (SO) in Fig. 2S of the ESI† within the GGA+U/GGA+U+SOC scheme. In the case of the NM phase, both Os ions remain non-SP. In contrast, they are aligned (↑↑)/anti-aligned (↑↓) for the FM/FIM SO. It is demonstrated that the *E*_t_ of the FM SO is lower than that of the NM/AFM state in both methods (see Fig. 2S of the ESI†). However, it is experimentally observed that the system persists in a paramagnetic state for 600 K to 2 K temp. and there is no magnetic phase transition occurring.^45^ Additionally, the computed *E*_t_ values of the FM and FIM SO are compared to determine the MGS for the Ir-dop. SCOO DPO. In the case of the FM/FIM SO, Os and Ir ions, the spins remain parallel (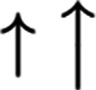)/anti-parallel (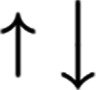) to each other, where the small and large arrow lengths represent their respective spin magnitudes. It is established that the FIM state is more stable than the FM one with an energy difference of Δ*E* = *E*_FIM_ – *E*_FM_ = −96 meV (see Table 2). This indicates that Ir and Os ion spins favor anti-alignment in both the in-plane and out-of-plane orientations. Hence, for further investigations only FM/FIM SO is considered for the prs./Ir-dop. motif.

**Table 2:** Computed total energy difference (Δ*E*), energy gap in the spin majority/minority channel (*E*^N↑^_g_/*E*^N↓^_g_), total/partial spin/orbital moment (*m*_t_/*m*_s_/*m*_orb._) in *μ*_B_, magnetocrystalline anisotropy energy (MAE) in meV, Curie temperature (*T*_C_) and MAE constant (*K*) per unit volume (×10^7^ erg cm^−3^) within the GGA+U/GGA+U+SOC method for the prs. and Ir-dop. Sr_2_CaOsO_6_ structures. The symbol M symbol represents the metal

Property	GGA+U				GGA+U+SOC			
	prs.		Ir-dop.		prs.		Ir-dop.	
Δ*E*	−51		−131.8		—		−96.4	
*E* ^N↑^_g_	0.048		1.15		0.004		M	
*E* ^N↓^_g_	1.86		M		—		—	
MAE	—		—		2.95		0.79	
*K*	—		—		1.72		0.46	
*T*_C_	185		171		—		—	
*m*_t_	4.0		1.0		3.84		0.91	
	**Os1**	**Os2**	**Os**	**Ir**	**Os1**	**Os2**	**Os**	**Ir**
*m*_s_	1.19	1.19	1.01	−1.39	1.09	1.09	0.89	1.25
*m*_orb._	—	—	—	—	−0.5	−0.5	0.44	0.083

**Fig. 4:**
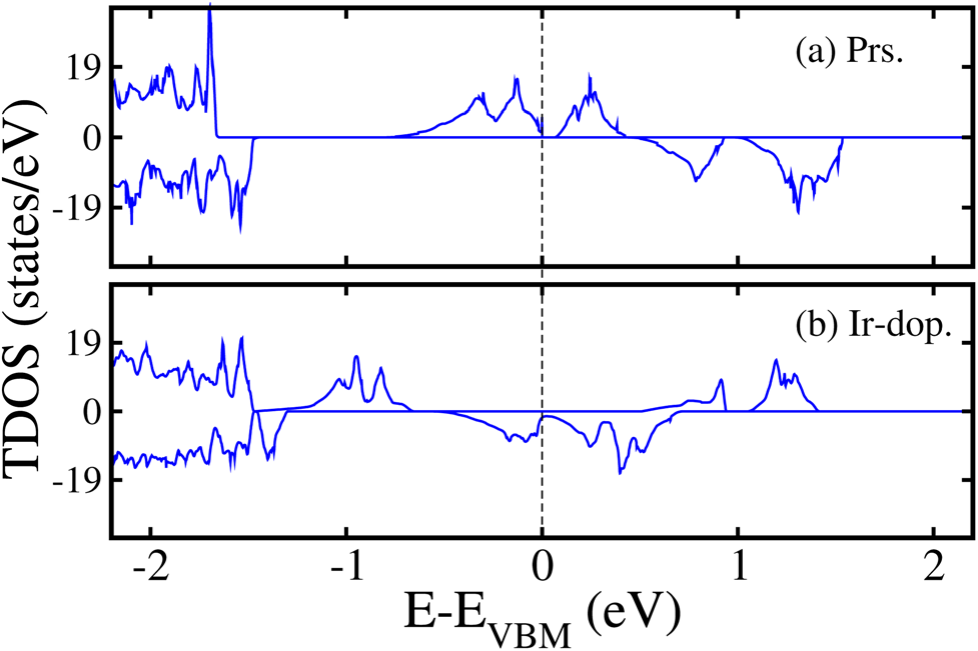
GGA+U computed non-degenerate total density of states (TDOS) in the (a) prs. and (b) Ir-dop. Sr_2_CaOsO_6_ structures.

Next, to examine the electronic structure of the prs./Ir-dop. SCOO system, we computed the total density of states (TDOS) in the stable FM/FIM SO within the GGA+U method. Fig. 4(a) shows that the prs. motif is SC with an *E*_g_ of 0.048 eV (as listed in Table 2). The remarkable finding of the present work is that a transition from the SC to HM state occurs in the Ir-dop. structure (see Fig. 4(b)), where a few states cross the Fermi level (*E*_F_) in the N^↓^, while it contains a definite large *E*_g_ of 1.15 eV in the N^↑^.

**Fig. 5:**
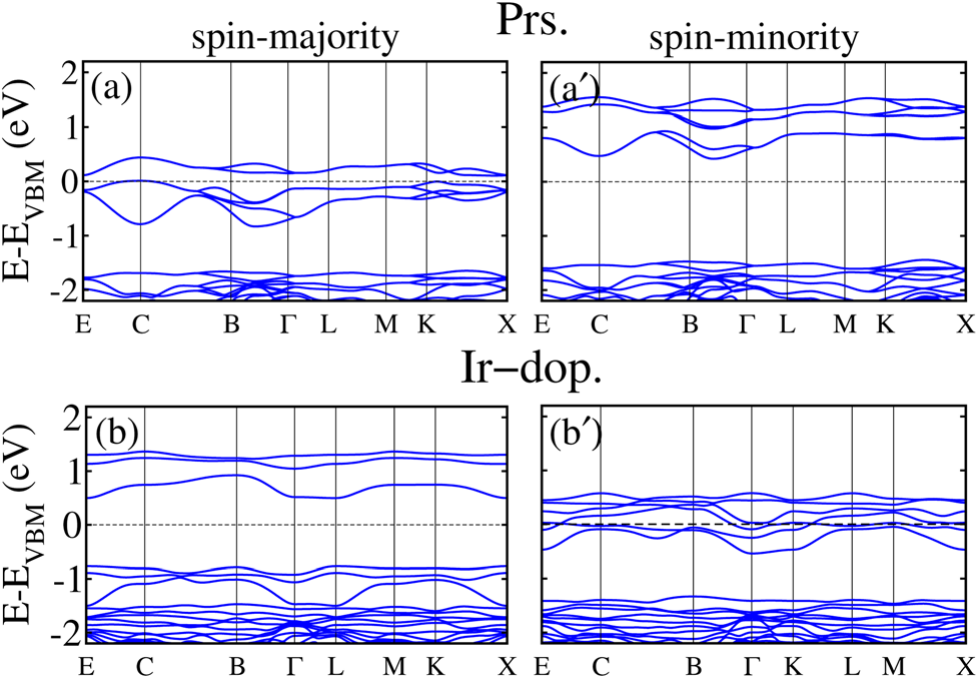
GGA+U computed non-degenerate spin-majority (left column)/spin-minority (right column) band structures for the (a and a′) prs. and (b and b′) Ir-dop. Sr_2_CaOsO_6_ structures.

Additionally, we presented the orbital resolved partial density of states (PDOS) on the 5d-states of the Os and Ir/Os ions in the prs. and Ir-dop. system in Fig. 3S of the ESI,† to better examine the states close to *E*_F_. The Os 5d states are dominant at the valence and the conduction band edges (VBE and CBE) as demonstrated in Fig. 3S(a) of the ESI.† As Os is in a +6(5d^2^) oxidation state, this results in the filling of two t_2g_ states in the N^↑^ and the e_g_ states are empty. Hence, the filled states are in the VB, while the two empty states shift towards the CB. For the Ir-dop. structure, metallicity in the N^↓^ mainly arises from the Os-5d states along with substantial contributions from Ir-5d states (see Fig. 3S(b) of the ESI†). It is found that the Os-5d states become partially occupied and significantly shift towards lower energies in the CB, which leads the system into the HM phase. This happens because the Ir ion is in a +4(5d^5^) oxidation state and it contributes 3 additional electrons to the system; one of the additional electrons occupies the t^↓^_2g_ and two occupy the t^↑^_2g_ state. Hence, they produce a repulsive force in the Os t_2g_ states, causing the Os states to move towards the CB from the VB in the N^↓^. Along with this, we plotted the computed non-degenerate band structures for both systems within the GGA+U scheme in Fig. 5 to provide further confirmation of their electronic states. The corresponding MI/HM behavior in the prs./Ir-dop. structure is well illustrated by Fig. 5(a, a′) and (b, b′), which also support the estimated TDOS in Fig. 4(a) and (b). Moreover, we plotted the GGA+U+SOC computed TDOS in Fig. 4S of the ESI† for the prs./Ir-dop. SCOO system in the most stable FM/FIM SO. A small *E*_g_ of 0.0045 eV exists in the prs. system (see Fig. 4S(a) of the ESI†), while the Ir-dop. motif turns metallic (see Fig. 4S(b) of the ESI†). It is predicted that the *E*_g_ computed within the GGA+U+SOC scheme for the prs. motif decreases (see Fig. 4S(a) of the ESI†) as compared to the GGA+U one (see Fig. 4(a)). This is because relativistic effects and SOC interactions often lower the *E*_g_ by mixing the orbitals in a way that reduces the energy splitting of the electronic states close to the *E*_F_.

**Fig. 6:**
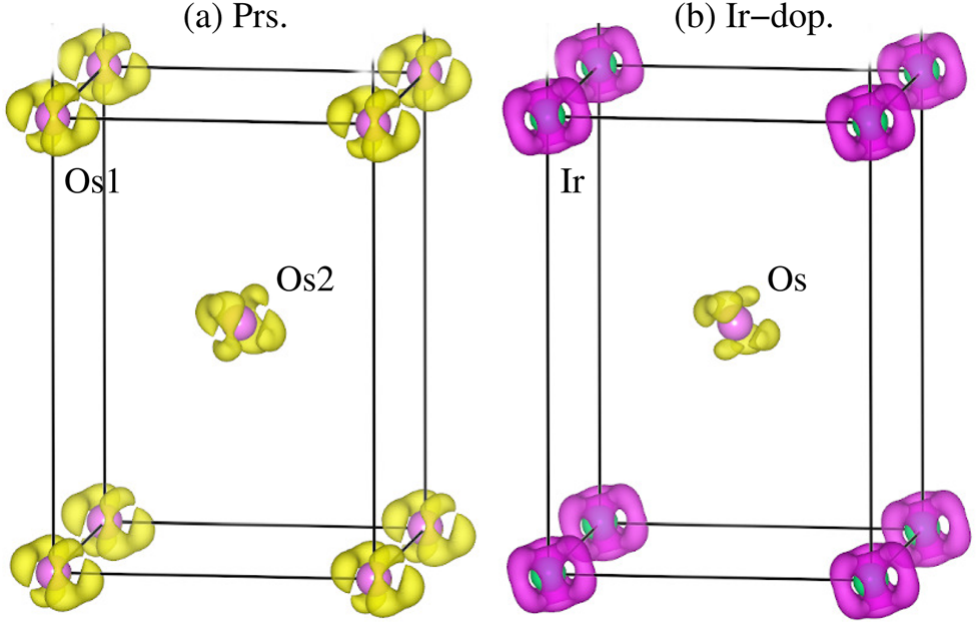
Computed spin magnetization density iso-surfaces for the (a) prs. and (b) Ir-dop. Sr_2_CaOsO_6_ structures with an iso-value of ±0.05% e Å^−1^.

Now, the system’s magnetism is described by calculating the total/partial spin magnetic moment (*m*_t_/*m*_s_) in each case along with the three-dimensional (3D) spin magnetization density isosurfaces. The estimated *m*_t_ for the prs./Ir-dop. system is 4.0/1.0*μ*_B_ f.u.^−1^ (see Table 2). The computed *m*_s_ for the Os1/Os2 ion in the prs. system is 1.19*μ*_B_, whereas the *m*_s_ on the Os/Ir is 1.01/−1.39*μ*_B_ for the Ir-dop. structure, as displayed in Table 2. The “−” sign shows that Ir and Os ions’ *m*_s_ are aligned antiparallel (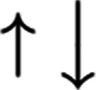) to each other, which indicates that an AFM coupling is dominant, and turns out to be a FIM SO in the Ir-dop. structure. Further, the computed *m*_s_ on the Os/Ir assures that they are in a +6(t^2^_2g_↑t^0^_2g_↓e^0^_g_↑e^0^_g_↓)/+4(t^3^_2g_↑t^2^_2g_↓e^0^_g_↑e^0^_g_↓) state. Moreover, the computed *m*_orb._ on the Os is −0.5*μ*_B_ in the prs. and 0.44/0.08*μ*_B_ on the Os/Ir in the Ir-dop. structure. Also, the 3D spin magnetization density isosurfaces, keeping an iso-value of ± 0.05% e Å^−1^, are plotted in Fig. 6 for the direct observation of *m*_s_ as well as to further affirm the SO in both structures. An appropriate density arises around the Os ions in the prs. system (see Fig. 6(a)), where the same density colors confirm that their spins’ parallel (↑↑) alignment and t^2^_2g_ orbital characterization is visualized due to its +6 state. Meanwhile in the Ir-dop. motif, the magnitude of density on the Ir ion is greater than that on the Os ion, which further verifies the magnitude of the calculated *m*_s_ on each ion. Besides this, the color contrast of the densities also assures that the spins of both ions align antiparallel (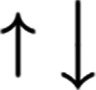) to each other.

**Fig. 7:**
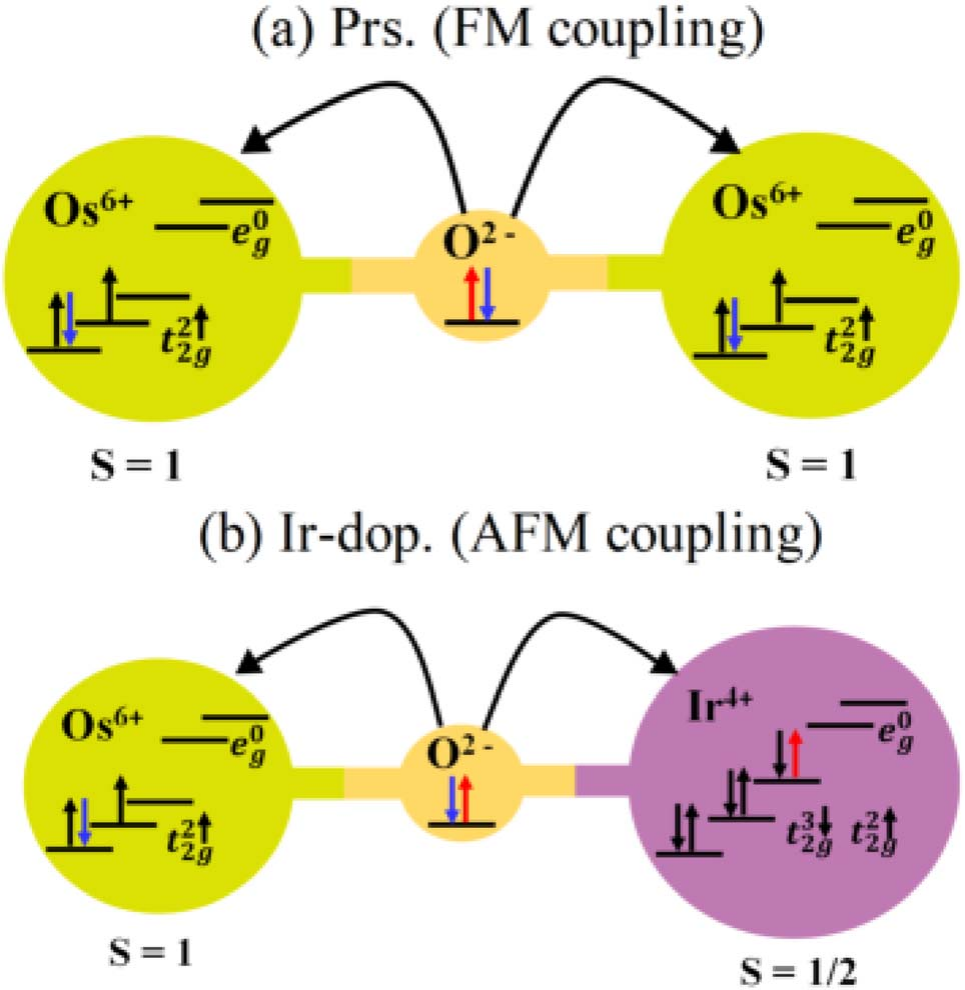
The superexchange interaction process between (a) Os^+6^ 5d and Os^+6^ 5d *via* oxygen results in a ferromagnetic (FM) coupling in the prs. and that between (b) Ir^+4^ 5d and Os^+6^ 5d *via* oxygen results in a strong antiferromagnetic (AFM) coupling in the Ir-doped Sr_2_CaOsO_6_ structure, which leads to ferromagnetic ordering.

Now, the FM SO in the prs. structure is explained by virtual hopping between less-than-half-filled d-orbitals *via* oxygen (Os^+6^–O^−2^–Os^+6^), as depicted in Fig. 7(a). Since the B-site atom is a non-magnetic cation, the magnetic aspects are determined by exchange coupling within the Os^+6^ ion, and a FM superexchange interaction occurs at 180°. But in the Ir-dop. system, the electron hopping takes place between the half-filled d-orbitals of Os^+6^ and half-filled d-orbitals of the Ir^+4^ ion, which occurs 180° between Os and Ir ions as Os^+6^–O^−2^–Ir^+4^ (see Fig. 7(b)). This supports the strong AFM superexchange mechanism in this structure, resulting in the FIM SO. Now, it is a well-established fact that MAE plays an important part in defining coercivity, which is required for permanent magnets with long-term magnetization.^56,57^ Therefore, we investigated the MAE and MAE constant 
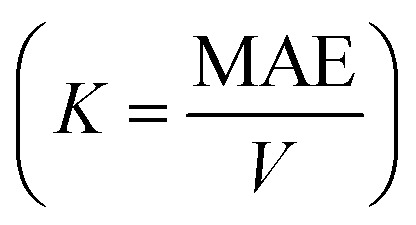
 as well as its relation to the structural distortions. To do this, we set the magnetization direction along the [001]/[010]/[100]-axis and compare *E*_t_. Moreover, a dense *k*-mesh of 12 × 12 × 9 is utilized to verify that the *E*_t_ and MAE values converged correctly. Our results reveal that the prs. system has a MAE/*K* of 2.95 meV/1.72 × 10^7^ erg per cm^3^ with an easy axis of [100] and average ∠Os–O–Os of 153.2° In comparison, the Ir-dop. system has a lower MAE/*K* of 0.79 meV/0.42 × 10^7^ erg per cm^−3^, as listed in Table 2, with an easy axis of [010] and an average ∠Os–O–Ir of 153.6°. This clearly illustrates that the MAE value is highly dependent on the structural distortions as reported in previous works.^5,21^

Next, to evaluate the *T*_C_ of the prs./Ir-dop. SCOO motif, the exchange constants (*J*) are determined using the Heisenberg model, as follows:^58^3
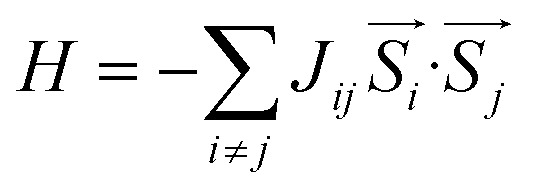
where 
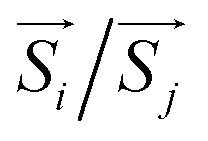
 specifies the spin vectors and can be calculated as *S*(*S* + 1)^1/2^. The *S* indicates the *m*_s_ on each magnetic ion, where *J* respresnts the interactions between inos. The *E*_t_ of FM, FIM, and AFM SO can be written as:

4 *E*^FM^_t_ = *E*_0_ + (*S*_1_(*S*_1_ + 1))^1/2^(*S*_2_(*S*_2_ + 1))^1/2^(2*J*_1_ + 4*J*_2_)

5 *E*^FIM^_t_ = *E*_0_ + (*S*_1_(*S*_1_ + 1))^1/2^(*S*_2_(*S*_2_ + 1))^1/2^(−2*J*_1_ + 4*J*_2_)

6 *E*^AFM^_t_ = *E*_0_ + (*S*_1_(*S*_1_ + 1))^1/2^(*S*_2_(*S*_2_ + 1))^1/2^(−2*J*_1_ − 4*J*_2_)

*E*_0_ refers to the energy in the spin-degenerate system and *S*_1_(*S*_2_) = 1(1) for Os_1_^+6^/Os_2_^+6^ in the prs. system and 
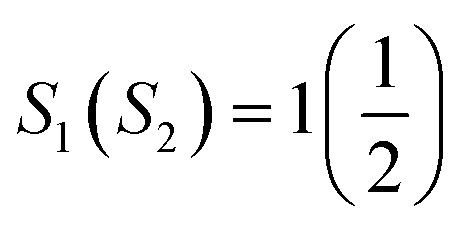
 for Os^+6^(Ir^+4^) in the Ir-dop. motif. Hence, *T*_C_ can be determined as follows:^59^7
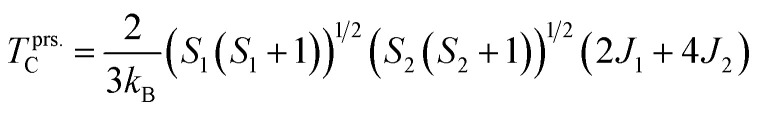
8
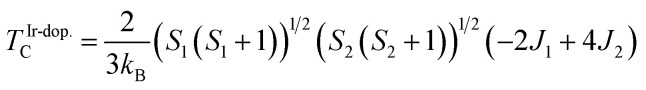
Our results revealed that the determined *T*_C_ is 185/171 K for the prs./Ir-dop. motif, as listed in Table 2. As structural distortions are reduced in the Ir-dop. system as compared to the prs. one, this lowers the *T*_C_ a bit, which is also consistent with the previous reports that *T*_C_ decreases with a reduction in structural distortion.^5^

**Fig. 8:**
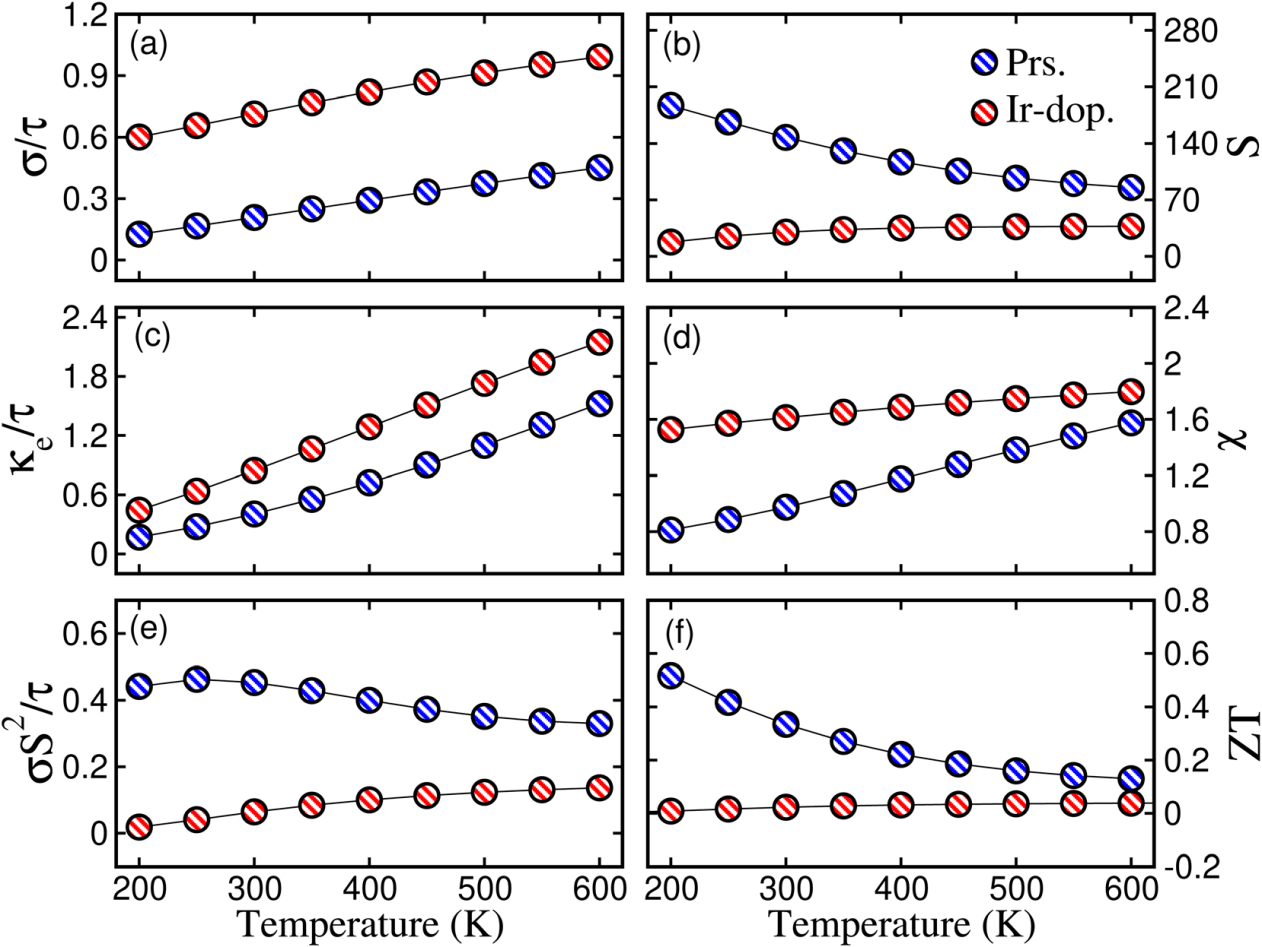
Computed (a) electrical conductivity per relaxation time 
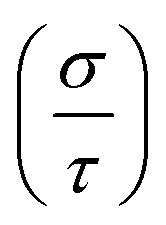
 in × 10^19^ Ωm^−1^ s^−1^, (b) Seebeck coefficient (*S*) in μV K^−1^, (c) thermal conductivity per relaxation time 
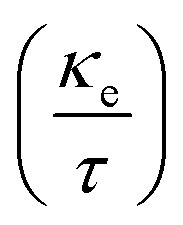
 in × 10^14^ W m^−1^ K^−1^ s^−1^, (d) susceptibility (*χ*) in × 10^−9^ m^3^ mol^−1^, (e) power factor (PF) in × 10^11^ W m^−1^ K^−2^ s^−1^, and (f) figure of merit (*ZT*) for the prs./Ir-dop. Sr_2_CaOsO_6_ structure.

Next, TE parameters are computed to provide insight into these materials for their potential realization in devices that convert heat into electricity. The BoltzTrap code^44^ is used to calculate the TE factors, such as the electrical conductivity (*σ*) per relaxation time 
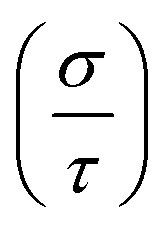
, Seebeck coefficient (*S*), electronic thermal conductivity (*κ*_e_) per relaxation time 
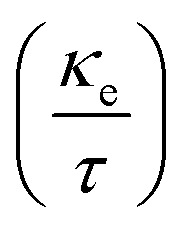
, susceptibility (*χ*), power factor 
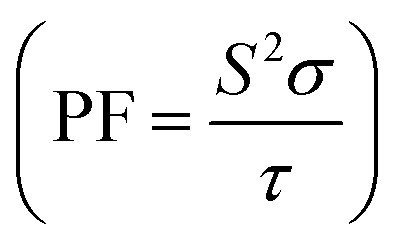
, and figure of merit 
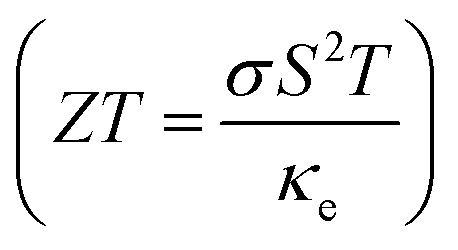
 at temp. ranging from 200 to 600 K, as presented in Fig. 8. As efficient TE materials require a high value of 
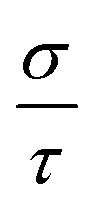
, the variation in temp.-dependent 
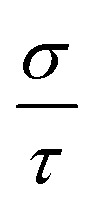
 is illustrated in Fig. 8(a); it depends on the concentration of the free carriers (electrons or holes) and increases with temp. due to an increase in kinetic energy. The values of 
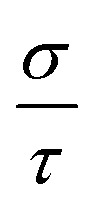
 are higher for the Ir-dop. motif, because there is a greater concentration of free charge carriers at *E*_F_. Its value is 0.21/0.71 × 10^19^ Ωm^−1^ s^−1^ at 300 K and reaches 0.45/0.9 × 10^19^ at 600 K for the prs./Ir-dop. structures. The *S* value, which is another important TE factor for determining the electronic transport properties, measures the TE capacity of a material to generate a potential difference across its edges as a result of a gradient. The temp. difference supports the passage of carriers, creating a gradient that can be determined in μV K^−1^. Fig. 8(b) demonstrates that *S* varies significantly between 30 to 200 μV K^−1^ for the considered temp. range. The computed *S* values for the prs./Ir-dop. structure are 147/30 μV K^−1^ and 85/37 μV K^−1^ at 300 K and 600 K, respectively. Hence, one can conclude from Fig. 8(a) and (b) that the behavior of *S* against temp. is in contrast to that of 
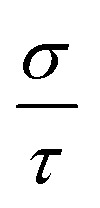
: whereas *S* decreases with temp., 
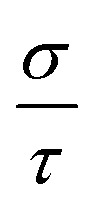
 increases. In addition to 
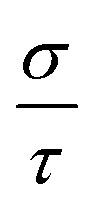
, Fig. 8(c) displays 
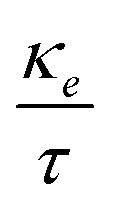
, which shows an increase with temp., being 0.41/0.84 × 10^14^ at 300 K and 1.6/2.1 × 10^14^ W m^−1^ K^−1^ s^−1^ at 600 K for the prs./Ir-dop. system. Next, the term *χ* describes the response of the TE materials to variations in external conditions such as electric or magnetic fields, which may have an impact on their TE performance. A higher temp. can enhance the TE performance of a material because the thermal energy pushes more charge carriers into the CB, increasing sensitivity to external forces. Hence, Fig. 8(d) exhibits that *χ* increases with the rise in temp. for the prs./Ir-dop. structure, from 0.9/1.6 × 10^−9^ m^3^ mol^−1^ to 1.5/1.8 × 10^−9^ m^3^ mol^−1^. Similarly, the power factor (PF) is also an important factor to consider when measuring a material's TE efficiency. The trend of the PF is like that of *S*, which decreases with an increase in temp. The calculated value of PF at 300 K is 0.45/0.064 × 10^11^ W m^−1^ K^−2^ s^−1^ and at 600 K is 0.32/0.13 W m^−1^ K^−2^ s^−1^ for the prs./Ir-dop. system (see Fig. 8(e)). Likewise, *ZT* is a critical component in determining TE efficiency for practical applications. The computed value of *ZT* at 300 K is 0.33/0.02 and at 600 K is 0.13/0.038 for the prs./Ir-dop. system (see Fig. 8(f)).

The net thermal conductivity (*κ*) is calculated as follows:

9 *κ* = *κ*_e_ + *κ*_l_

Slack's equation^60^ was used to calculate *κ*_l_ as follows:10
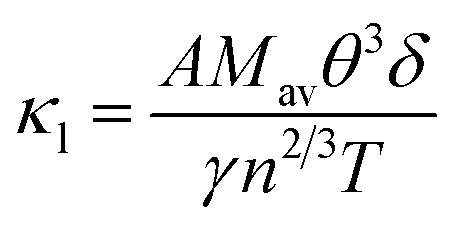
*M*_av_, *δ*, *n*, *T*, *γ*, and *θ* represent the average atomic mass in the crystal, cubic root of the average atomic volume, total number of atoms in the unit cell, absolute temp., Grüneisen parameter, and Debye temp., respectively. The Grüneisen parameter is determined *via* Poisson's ratio:11
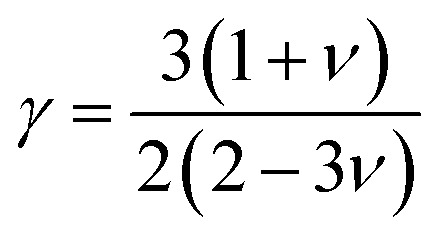
where *A* is calculated as follows:^61^12
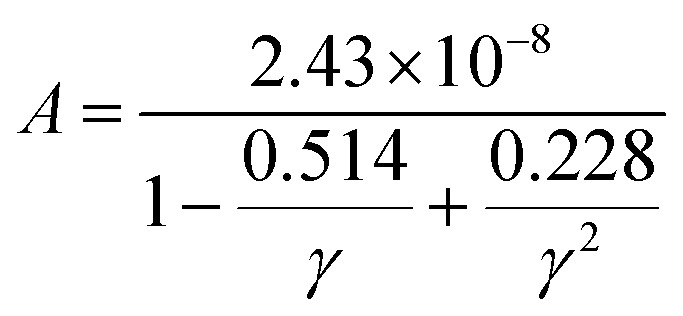
while *θ* can be determined as follows:^62^13
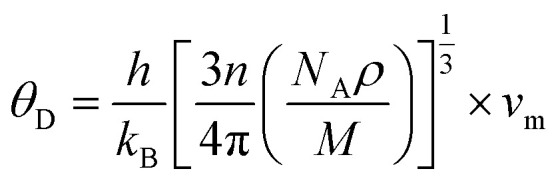
where *h*, *k*_B_, *n*, *N*_A_, *ρ*, *M*, and *v*_m_ serve as Planck’s constant, the Boltzmann constant, the total number of atoms per unit cell, Avogadro's number, density, molecular weight, and sound velocity, respectively. The average sound velocity (*v*_m_) is figured out using14
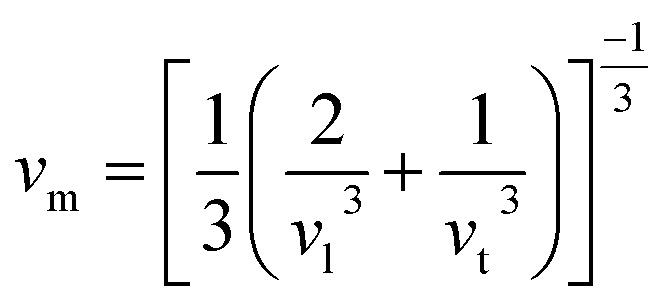
Here, *v*_l_/*v*_t_ is the longitudinal/transverse velocity, which can be measured as follows:15
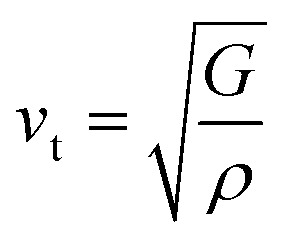
16
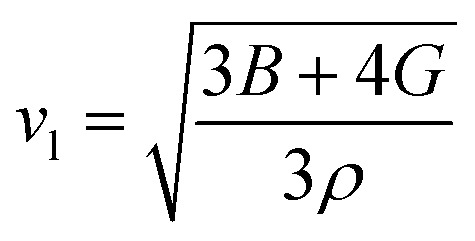


**Fig. 9:**
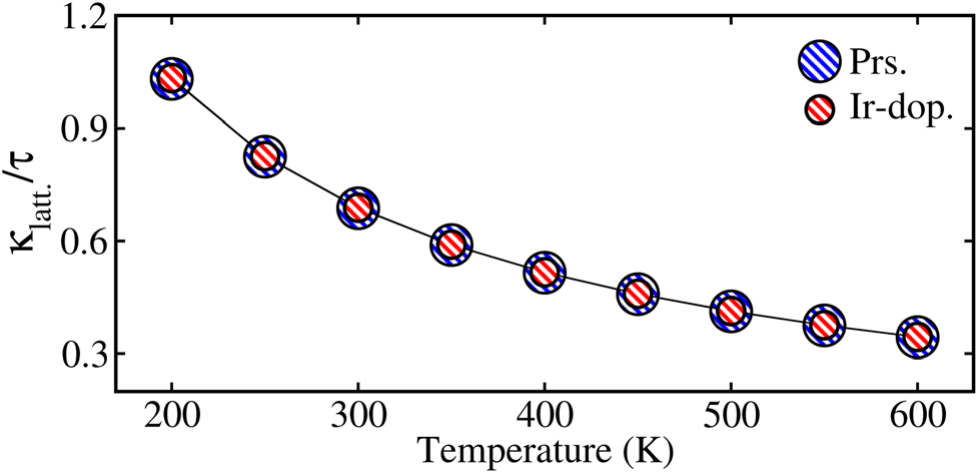
Computed values of lattice thermal conductivity per relaxation time 
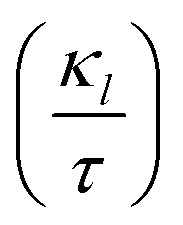
 in × 10^14^ W m^−1^ K^−1^ with temperature, for the prs./Ir-dop. Sr_2_CaOsO_6_ structure.

Fig. 9 shows that the estimated 
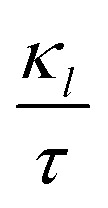
 decreases with increasing temp., which is a desired property for TE applications. The decrease in 
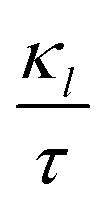
 as temp. rises is due to increased phonon scattering caused by higher lattice vibrations, which decreases the mean free route of phonons. The established values of 
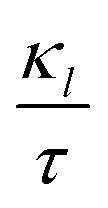
 for the prs. and Ir-dop. systems are 0.68 and 0.69 × 10^14^ W m^−1^ K^−1^, respectively.

Here, we would like to mention that the Slack method predominantly considers acoustic phonon contributions, disregarding optical phonon modes, and complicated anharmonic interactions, which may be crucial in materials characterized by poor symmetry or complicated bonding environments. Consequently, the model may generate considerable error in the calculation of *κ*_l_, thereby impacting the precision of resultant TE parameters including *ZT*. Recent investigations have underscored the ambiguities linked to the Slack model. For example, Rabin *et al.*^63^ shown that the Slack formula often overestimates *κ* in materials characterized by low group velocities and substantial optical phonon contributions, which are particularly influential in materials exhibiting poor symmetry or intricate bonding environments. Consequently, the model may generate significant error in the calculation of *κ*_l_, possibly compromising the precision of derived thermoelectric parameters, including the *ZT*. Likewise, J. Carrete *et al.*^64^ also noted that the empirical coefficient in the Slack equation is not universal as it changes according to the Grüneisen parameter and the bonding properties of the material. Hence, inaccuracies in *κ*_l_ can influence the assessment of the thermoelectric *ZT*, either under-representing or overstating a material’s potential, thereby impacting the precision of derived thermoelectric parameters such as the *ZT*. The Slack model provides a valuable first estimate. However, enhanced accuracy in predictions can be attained by resolving the Boltzmann transport equation for phonons using first-principles interatomic force constants. In future endeavors, we intend to integrate sophisticated methodologies to authenticate and enhance the outcomes derived from the empirical methodology.

Usually, the empirical Slack model offers a useful initial estimate of *κ*, but it fails to adequately represent phonon transport dynamics in intricate materials. Recent developments in computational materials science have resulted in the emergence of first-principles approaches that offer a more precise and physically substantiated framework for forecasting thermal transport parameters. One technique involves solving the Peierls–Boltzmann transport equation (PBTE) alongside interatomic force constants derived from density functional perturbation theory (DFPT). These approaches consider both acoustic and optical phonons and comprehensively capture phonon–phonon scattering processes, allowing more accurate predictions across a diverse array of materials. Ma *et al.*^65^ and Lindsay^66^ examine the theoretical basis and practical application of these first-principles methodologies. Ma *et al.* emphasise the accomplishments and persistent difficulties in modeling heat transport using PBTE + DFPT, particularly in low-dimensional and disordered systems. Lindsay offers a comprehensive examination of the concept and illustrates its efficacy in precisely modeling *κ* across several crystalline materials.

## 4 Strained systems

Here, we examine the biaxial ([110]) strain consequences for the structural, mechanical, and dynamical stability of the SCOO system by optimizing the two-axis lattice parameters *a* and *b* in the range from −5% to +5. First, the thermodynamical stability of the systems is examined by calculating Δ*H*_f_ under applied strain, and the results are shown in Fig. 5S of the ESI.† It is clear that Δ*H*_f_ is negative within all strain ranges; however, it is more negative under tens. strains, which means that these structures are more stable than the comp. strained ones. Next, we determine the mechanical stability of the structures under strain; 13 independent elastic constants are listed in Table 1, which obey the Born criteria and confirm mechanical stability against strain. Further, Fig. 1S of the ESI† displays the *B*/*Y*/*G* values, which show that the prs. system under −5% comp. strain is stiffer and harder than the tens. strain ones. Additionally, we plotted the *B*/*G*, *C*_p_, and *ν* values of both systems under −5% and +5% strain in Fig. 2, which depicted that structures are ductile and show more strength for −5% comp. strain. Moreover, we computed the phonon dispersion curves under two maximum strains of ±5%, as shown in Fig. 6S of the ESI,† to ensure the dynamic stability of the motifs. This shows that no negative frequencies exist, indicating that the systems are also dynamically stable for the considered higher strain values. Subsequently, the magnetic ground state stability of the Ir-dop. SCOO system is examined by plotting Δ*E* = *E*_FIM_ – *E*_FM_ against strain, as shown in Fig. 7S of the ESI.† It is found that Δ*E* remains negative within all considered strain ranges, which indicates that FIM is more stable than the FM SO in the whole strain range.

**Fig. 10:**
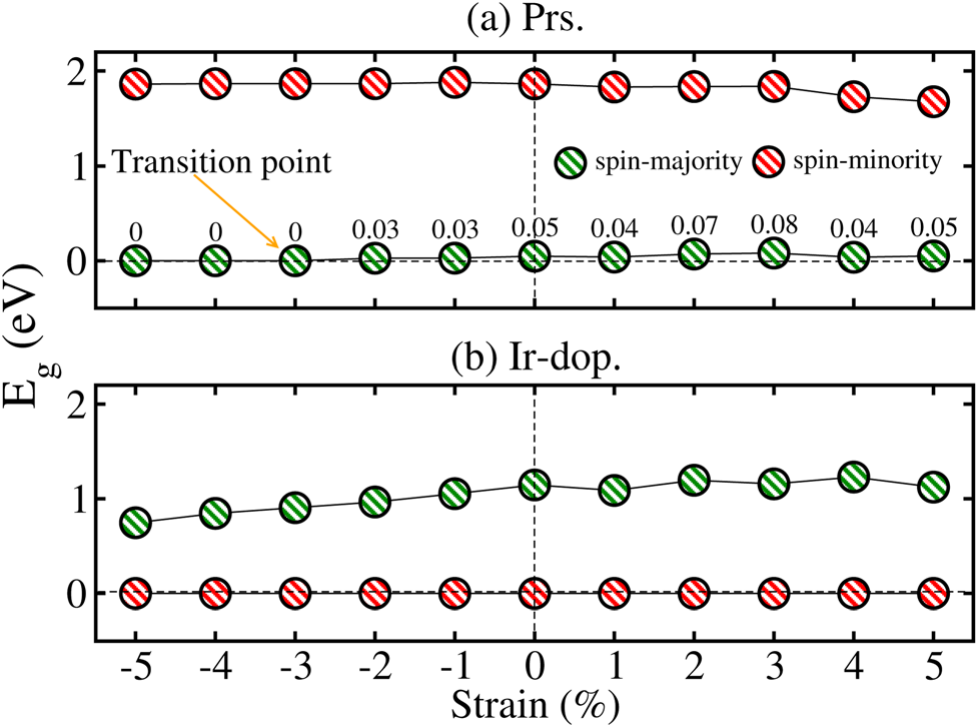
GGA+U calculated energy gap (*E*_g_) against ±5% biaxial ([110]) strain in the spin majority (N^↑^)/spin-minority (N^↓^) channel of the (a) prs. and (b) Ir-dop. Sr_2_CaOsO_6_ structures.

**Fig. 11:**
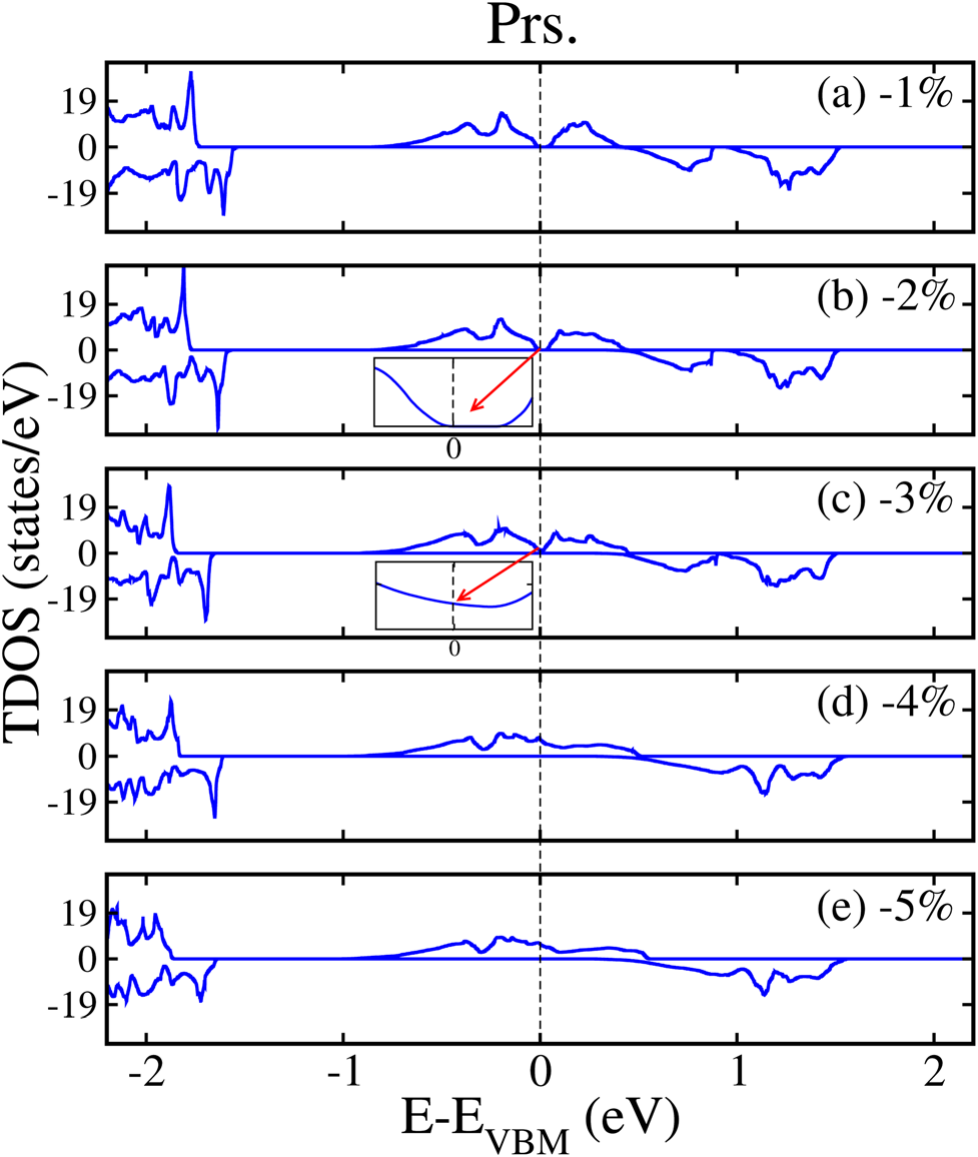
GGA+U computed non-degenerate total density of states (TDOS) of the prs. Sr_2_CaOsO_6_ structure for (a) −1%, (b) −2%, (c) −3%, (d) −4%, and (e) −5% biaxial ([110]) compressive strain.

To qualitatively describe how the electronic structure of the systems varies under strain, we plotted the computed *E*_g_ in the N^↑^ and N^↓^ against strain, as shown in Fig. 10. When tens. strain is varied from 0% to +5%, a slight linear increase in *E*_g_ from 0.048 to 0.050 eV in the N^↑^ is observed (see Fig. 10(a)) for the prs. system. Conversely, when comp. strain is varied from 0% to −2%, the computed *E*_g_ decreases from 0.048 to 0.027 eV and ultimately becomes zero at a crucial value of −3% in the N^↑^ (see Fig. 10(a)), while N^↓^ maintains a definite *E*_g_ for the whole strain range. Thus, the system exhibits a SC-to-HM transition at a crucial −3% strain and for higher comp. strains as well. Likewise, when the Ir-dop. system is subjected to strain from 0% to +5%, *E*_g_ decreases from 1.15 to 1.12 eV in the N^↑^ and decreases more significantly under comp. strain from 1.15 eV to 0.75 eV (see Fig. 10(b)). In contrast, N^↓^ preserves its metallic character for the whole strain range, which ensures the robustness of the HM phase in a doped system. In addition, we plotted the corresponding SP TDOS from −1% to −5% for the prs. system in Fig. 11, which also assures that no states lie at *E*_F_ and a small/large *E*_g_ exists in the N^↑^/N^↓^ for −1 and −2% strains (see Fig. 11(a) and (b)), correspondingly. However, the states overlap at the *E*_F_ in the N^↑^ and *E*_g_ becomes zero for a crucial −3% strain (see Fig. 11(c)), while a large *E*_g_ of 1.86 eV exists in the N^↓^. Hence, a SC-to-HM transition occurs at a crucial comp. strain of −3% for the prs. system. Moreover, N^↑^ becomes more metallic for the −4%/−5% strain (see Fig. 11(d) and (e)) as more electrons reside at *E*_F_ for these strain levels. Along with this, we plotted the SP TDOS for the tens. strain of +1% to +5% in the prs. system in Fig. 8S of the ESI,† which shows that no transition occurs at any strain value and a definite *E*_g_ exists in both channels. Hence, the system holds its insulating behavior for all tens. strain levels. Likewise, the calculated TDOS for the Ir-dop. under ±5% biaxial ([110]) strains is plotted in Fig. 9S of the ESI,† which is consistent with Fig. 10 and no significant changes occur with strain.

**Fig. 12:**
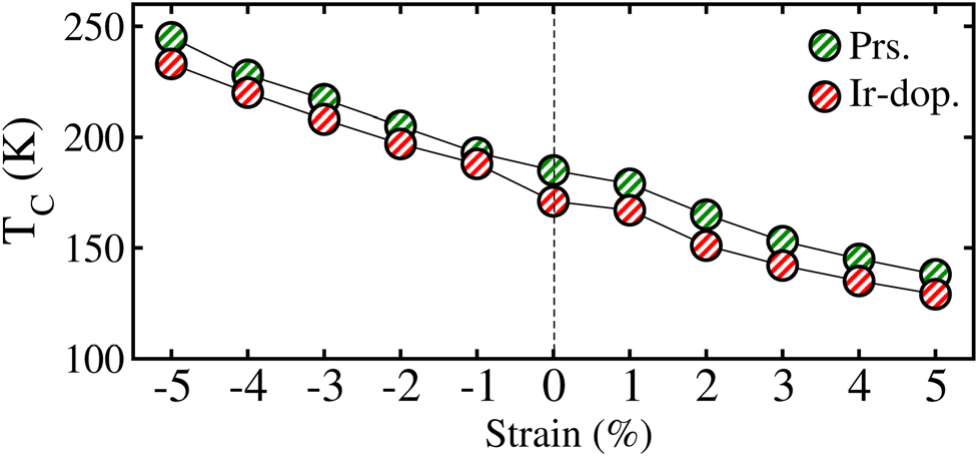
Computed Curie temperature (*T*_C_) under ±5% biaxial ([110]) strain for the prs. and Ir-dop. Sr_2_CaOsO_6_ structures.

Next, we investigated the strain-induces changes in the systems’ magnetism. To do this, the *m*_s_ on the Os/Ir ion in the prs. and Ir-dop. SCOO motifs is plotted in Fig. 10S of the ESI.† As the strain is varied from −5% to +5%, a slight change in the *m*_s_ value occurs in both systems. The *m*_s_ on the Os ion varies from 1.12*μ*_B_ to 1.18*μ*_B_ as strain changes from −5% to +5%. Within a similar strain limit, the *m*_s_ on Os/Ir changes from 0.9 to 0.95*μ*_B_/−1.35 to −1.32*μ*_B_ in the Ir-dop. case. Along with this, we computed *T*_C_ under ±5% biaxial ([110]) strain, as presented in Fig. 12 for both systems. It shows that under −5% strain, prs./Ir-dop. has the highest *T*_C_ of 245/233 K, while +5% strain gives the lowest *T*_C_ of 138/129 K. Under comp./tens. strain, structural distortions increase/decrease, which has a positive/negative effect on the *T*_C_ values, as discussed in previous theoretical works.^5,39^

**Fig. 13:**
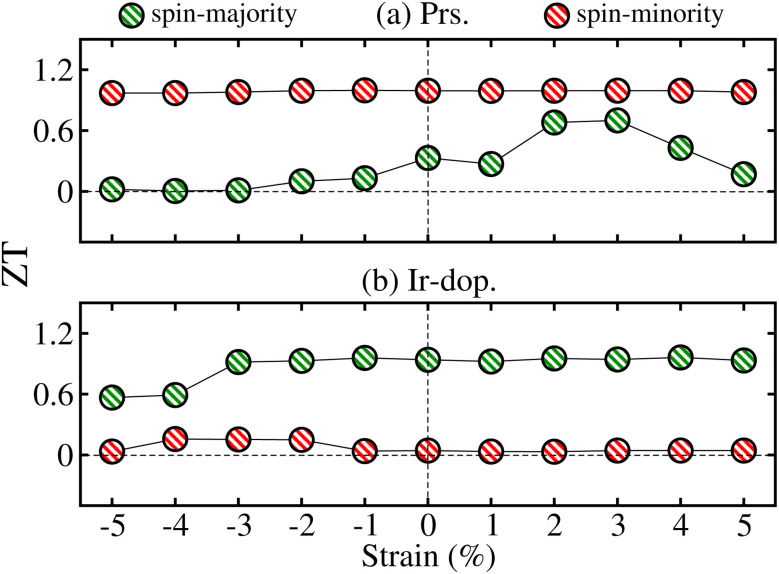
Computed figure of merit (*ZT*) in the spin-majority and spin-minority channels for the (a) prs. and (b) Ir-dop. Sr_2_CaOsO_6_ structures as a function of ±5% biaxial ([110]) strain.

Panneerselvam *et al.*^67^ found that altering the polar optical phonon scattering mechanism *via* strain in ScN significantly influences the variance in lattice thermal conductivity while minimally affecting the TE power factor values. Likewise, Zhang *et al.*^68^ examined the impact of strain on two-dimensional materials, emphasizing that strain engineering can improve TE capabilities by altering the electronic structure. Additionally, Yu *et al.*^69^ showed that strain engineering may markedly influence the *κ*_l_ and heat flow in Bi_2_Te_3_ nano-films, therefore highlighting the significance of strain in TE materials. The *κ*_l_ of the Bi_2_Te_3_ nano-film may be efficiently adjusted by the application of strain. A tensile strain of 6% can decrease heat conductivity by 50%; however, a compressive strain of 4% can enhance *κ*_l_ by 60%. Hence, we plotted the *ZT* values of N^↑^/N^↓^ at room temp. (300 K) as a function of ±5% strain in Fig. 13 for both systems. The total *ZT* is similar in the N↑/N↓ for the prs./Ir-dop. system because it is closely related to the real *E*_g_ value, in contrast to Fig. 10 (see Fig. 4). Therefore, we compare the *ZT* values with the calculated *E*_g_ (see Fig. 10) for the strained system, which reveals that *ZT* is substantially correlated with *E*_g_, where *ZT* increases as *E*_g_ increases and *vice versa*. The computed *ZT* under +2/+3% tens. strain is 0.68/0.71 for the prs. and 0.04/0.15 for the Ir-dop. system at 300 K. Therefore, we computed the TE factors of the prs. system under +2% and +3% biaxial tens. strains, as shown in Fig. 11S of the ESI.† As *E*_g_ increases under +2/+3% tens. strain, the values of 
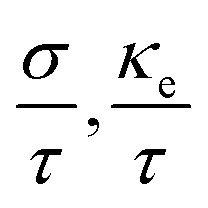
, and *χ* are less than those of the unstrained system because there is a smaller number of charge carriers across the *E*_F_, but the trend remains the same with temp. as compared to the unstrained system. Also, the values of the PF at +2/+3% strain are less than that of the unstrained system, whereas the values of *S* also increase at +2/+3% tens. strain and it follows the same trend with temp. as followed in the unstrained system. Besides this, we calculated the 
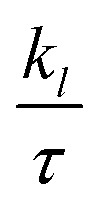
 of the prs. system under +2/+3% tens. strain, as presented in Fig. 12S of the ESI.† This shows that its value decreases with an increase in temperature, which is a required criterion for TE applications.

## 5 Conclusion

In summary, *ab initio* calculations were performed to examine the thermodynamic, mechanical, dynamical stability, thermoelectric, electronic structure, and magnetic properties of the unstrained and strained systems of the pristine (prs.)/Ir-doped (dop.) Sr_2_CaOsO_6_. It is predicted that both systems are thermodynamically and mechanically stable as they have negative formation energies and follow the Born criteria. Due to non-negative phonon frequency curves, they are dynamically stable as well. The unstrained prs./Ir-dop. system has a ferromagnetic (FM)/ferrimagnetic (FIM) ground state due to FM/antiferromagnetic (AFM) interactions between Os and Os/Os and Ir ions. The prs. motif is a semiconductor, while Ir-doping results in a half-metallic state (HM). The additional electrons produced by the dopant (Ir) cause a repulsion in the half-filled Os t^2^_2g_ in the spin-minority channel, which moves the Os bands towards the Fermi level, resulting in conductivity. Also, a large energy-gap of 1.15 eV in the non-metallic channel ensures the stability of the HM state of the system. Moreover, the computed *ZT* is 0.33/0.02 at 300 K for the prs./Ir-dop. motif. Further, our results indicate that the prs. system becomes half-metallic at a crucial value of −3% compressive strain. In contrast, the Ir-dop. motif retains its half-metallic nature against ±5% biaxial ([110]) strain. The prs./Ir-dop. structure has a high magneto-crystalline anisotropy energy (MAE) and MAE constants (*K*) of 2.95/0.79 meV and 1.72/0.46 × 10^7^ erg per cm^3^ at the Curie temperature of 185/171 K, respectively. Interestingly, *ZT* values increase in the prs. system under +2/+3% tens. strain because the energy gap increases against these strain values. Hence, near-unity *ZT* values suggest that these materials are very suitable for magnetic memory devices and thermoelectric applications.

## Data availability

The datasets used and/or analyzed during the current study are available from the corresponding author on reasonable request.

## Author contributions

Samia Shahzadi: writing – original draft, investigations, formal analysis, data curation. Ihab Mohamed Moussa: validation, visualization, resources, formal analysis, funding. Sohail Mumtaz: visualization, validation, formal analysis. S. Nazir: writing – review and editing, validation, supervision, project administration, conceptualization.

## Conflicts of interest

The authors declare no competing interests.

## Supplementary Material

RA-015-D5RA02453F-s001
