# Supplementary material for: Structural stabilities, robust half-metallicity, magnetic anisotropy, and thermoelectric performance of the pristine/Ir-doped Sr2CaOsO6: strain modulations
Source: RSC Adv. 2025 May 21;15(22):17142–52. doi: 10.1039/d5ra02453f (PMC12094283; doi:10.1039/d5ra02453f)
Supplement: RA-015-D5RA02453F-s001 [file RA-015-D5RA02453F-s001.pdf]

Supplementary Information (SI) for RSC Advances.
This journal is © The Royal Society of Chemistry 2025

Supporting Information

Structural Stabilities, Robust Half-Metallicity, Magnetic Anisotropy, and Thermoelectric Performance in the Pristine/Ir-Doped Sr_2CaOsO_6: Strain Modulations

Samia Shahzadi[1], Ihab Mohamed Moussa[2], Sohail Mumtaz[3], and S. Nazir[1]*
[1]*Department of Physics, University of Sargodha, 40100 Sargodha, Pakistan*
[2]*Department of Botany and Microbiology, College of Science, King Saud University, P.O. Box 2455, Riyadh, 11451, Saudi Arabia and*
[3]*Department of Chemical and Biological Engineering, Gachon University, 1342 Seongnamdaero, Sujeong-gu, Seongnam-si 13120, Republic of Korea*

* Electronic address: `safdar.nazir@uos.edu.pk,Tel:$+$92-334-971-9060`

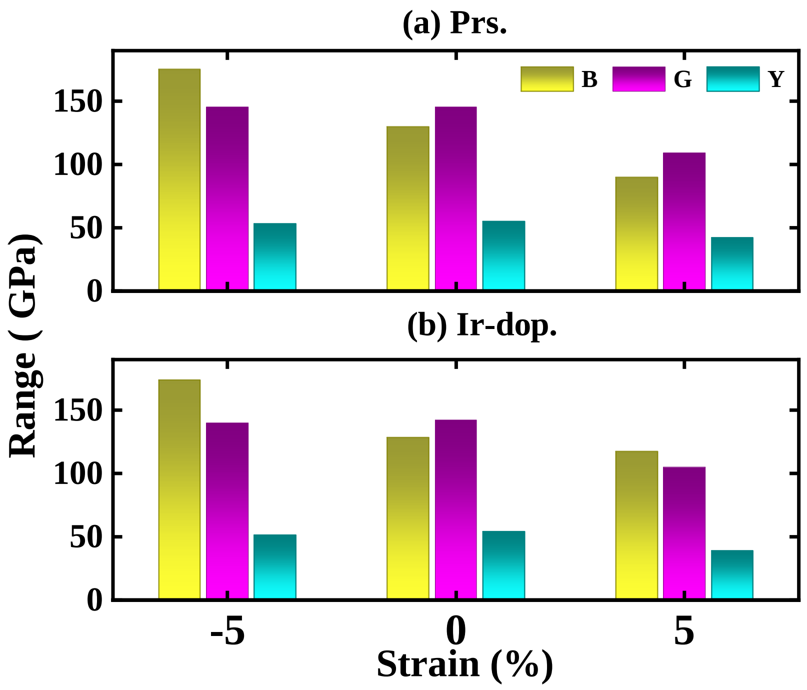

FIG. 1S: Computed Bulk modulus (B)/Shear modulus (G)/Youngs modulus (Y) for the (a) prs. and (b) Ir-dop. Sr_2CaOsO_6 double perovskite oxides.

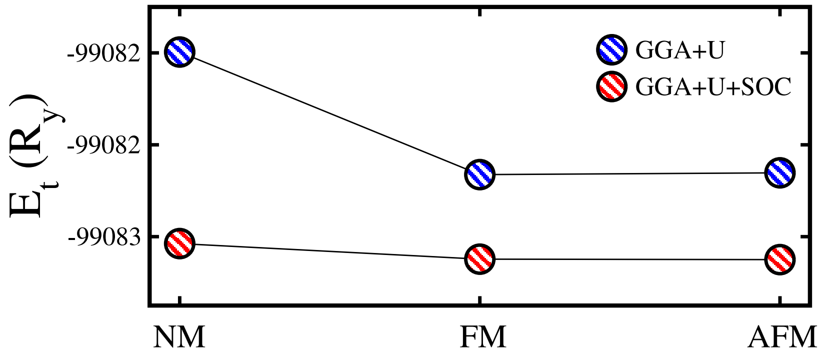

FIG. 2S: GGA+U/GGA+U+SOC computed total enrgy (E_t) in the non-magnetic (NM), ferromagnetic (FM), and antiferromagnetic (AFM) spin ordering in the prs. Sr_2CaOsO_6 double perovskite oxide.

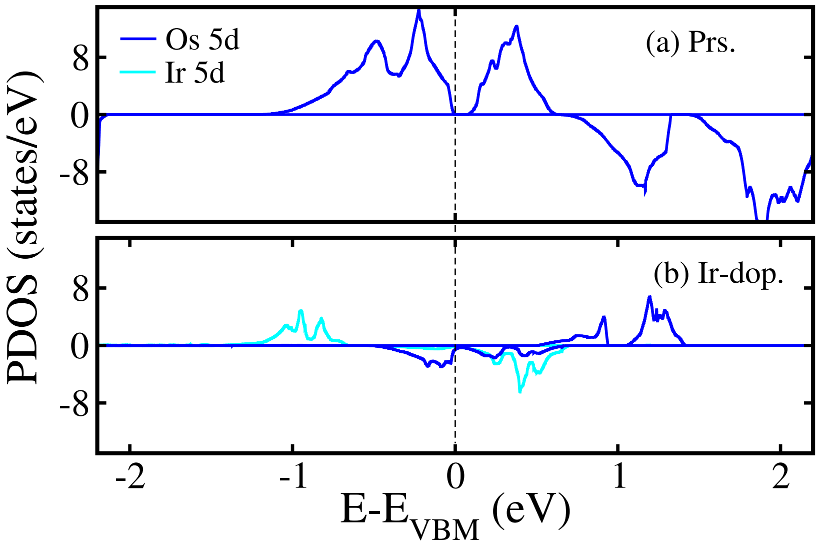

FIG. 3S: GGA+U computed non-degenerated $5d$ states resolved partial density of states (PDOS) projected on the Os/Ir ion in the (a) prs. and (b) Ir-dop. Sr_2CaOsO_6 double perovskite oxides.

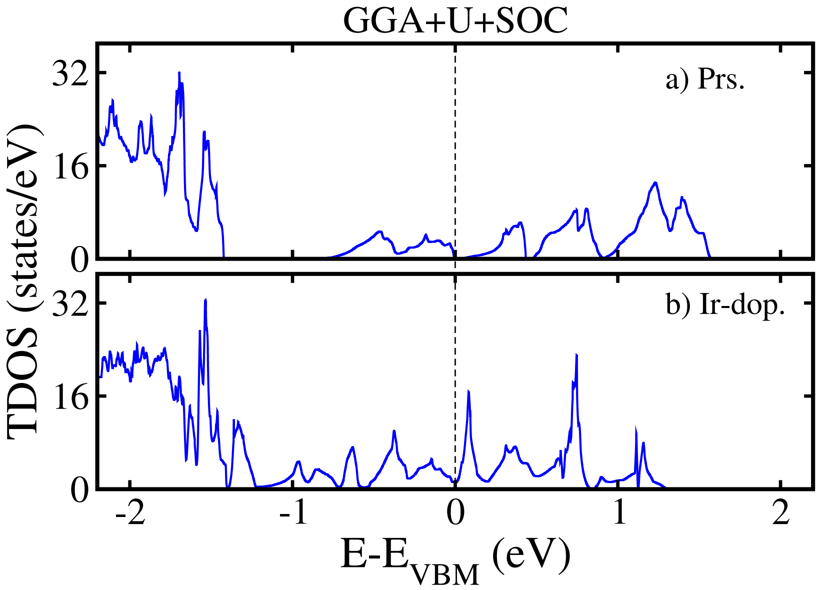

FIG. 4S: GGA+U+SOC computed non-degenerated total density of states (TDOS) in the (a) prs. and (b) Ir-dop. Sr_2CaOsO_6 double perovskite oxides.

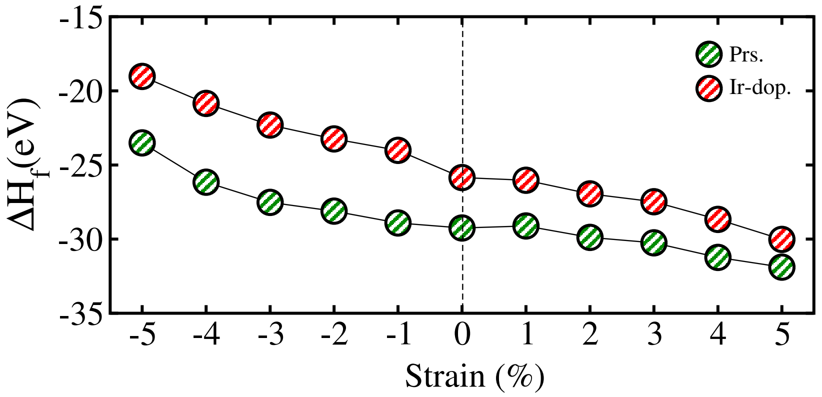

FIG. 5S: Computed formation enthalpy (ΔH_f) in the prs./Ir-dop. Sr_2CaOsO_6 double perovskite oxide under ±5% biaxial ([110]) strain.

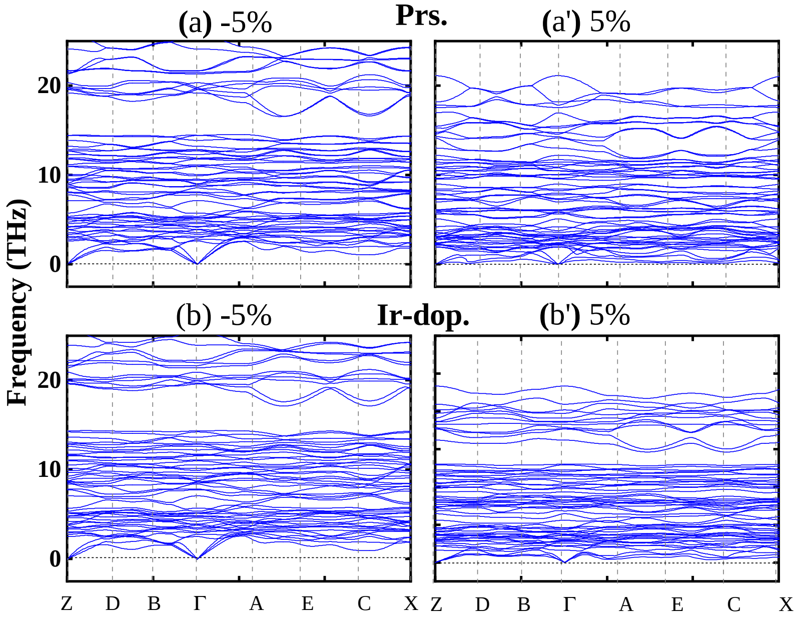

FIG. 6S: Computed phonon dispersion curves for the (a/b) −5% and (a′/b′) +5% biaxial strains ([110]) in the prs./Irs-dop. Sr_2CaOsO_6 double perovskite oxide.

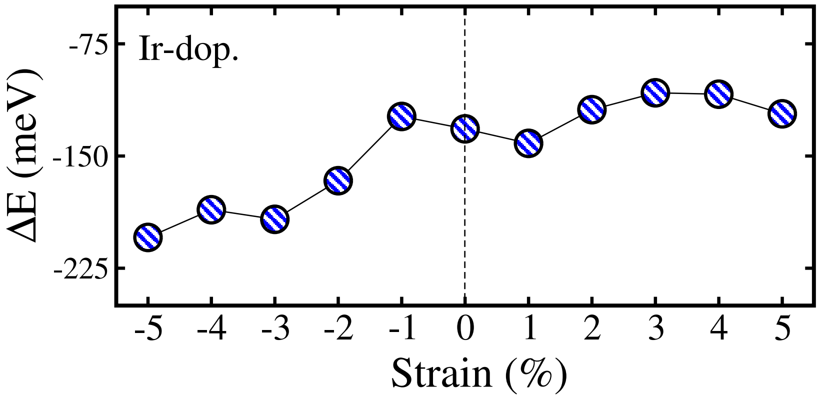

FIG. 7S: GGA+U computed energy difference ($\Delta E = E_{FIM} - E_{FM}$) of the stable state ferrimagnetic (FIM) and ferromagnetic (FM) of the Ir-dop. Sr_2CaOsO_6 double perovskite oxide as a function of ±5% biaxial ([110]) strain.

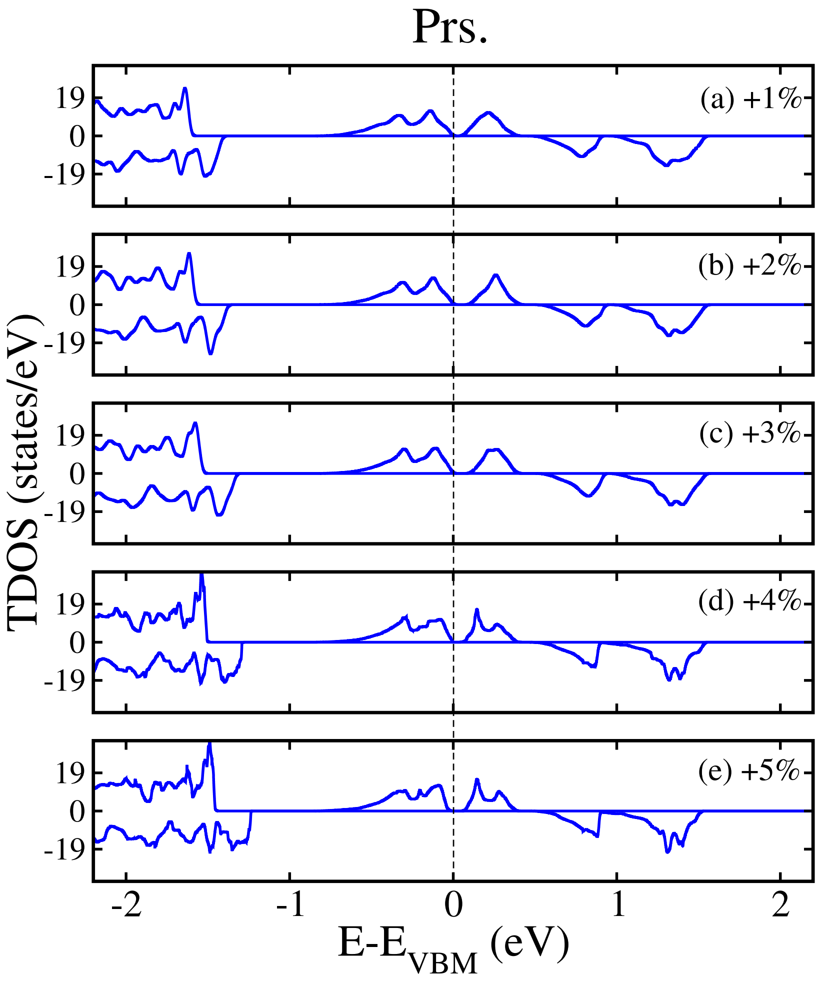

FIG. 8S: GGA+U Computed total density of states (TDOS) of the prs. Sr_2CaOsO_6 double perovskite oxide as a function of (a) +1%, (b) +2%, (c) +3%, (d) +4%, and (e) +5% biaxial ([110]) tensile strains.

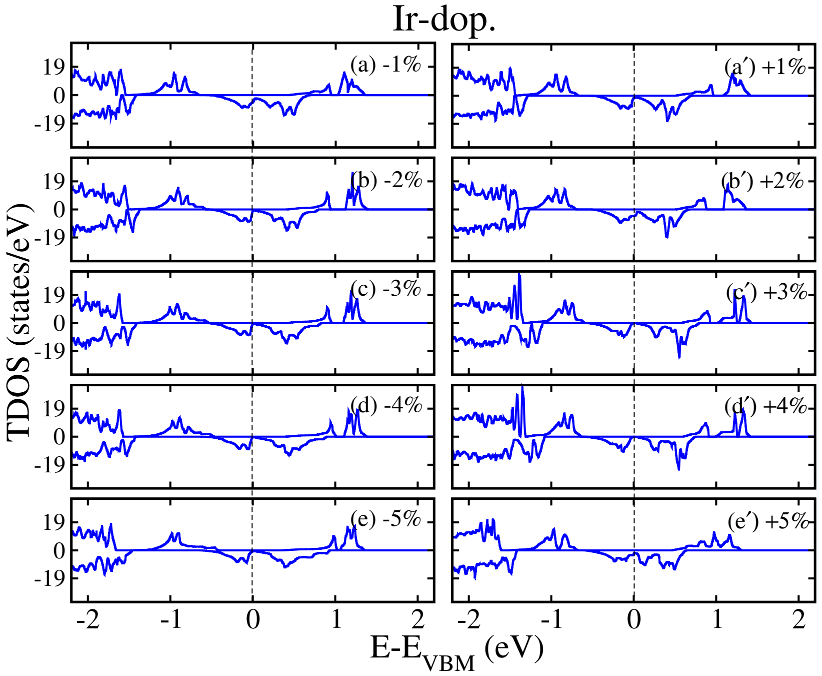

FIG. 9S: GGA+U computed total density of states (TDOS) of the Ir-dop. Sr_2CaOsO_6 structure for the (a/a′) −1/+1%, (b/b′) −2/+2%, (c/c′) −3/+3%, (d/d′) −4/+4%, and (e/e′) −5/+5% biaxial ([110]) compressive/tensile strains.

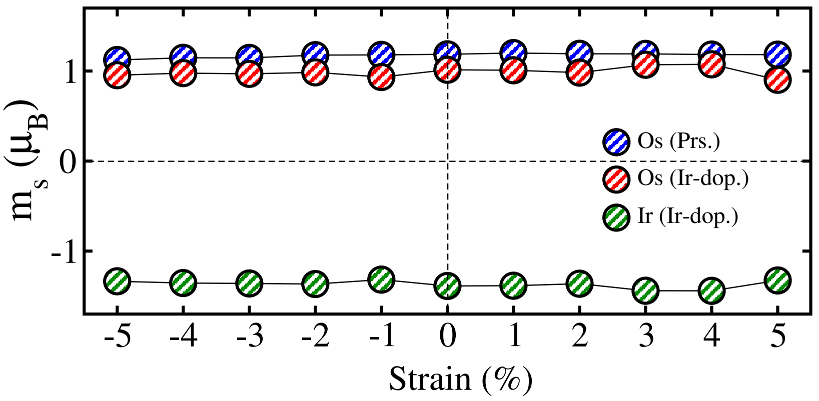

FIG. 10S: Computed partial spin magnetic moments (m_s) on the Os ion in the prs. and Os/Ir in the Ir-dop. (Ir-subsituted at the Os-site) in the Sr_2CaOsO_6 double perovskite oxide as a function of ± 5% biaxial ([110]) strain.

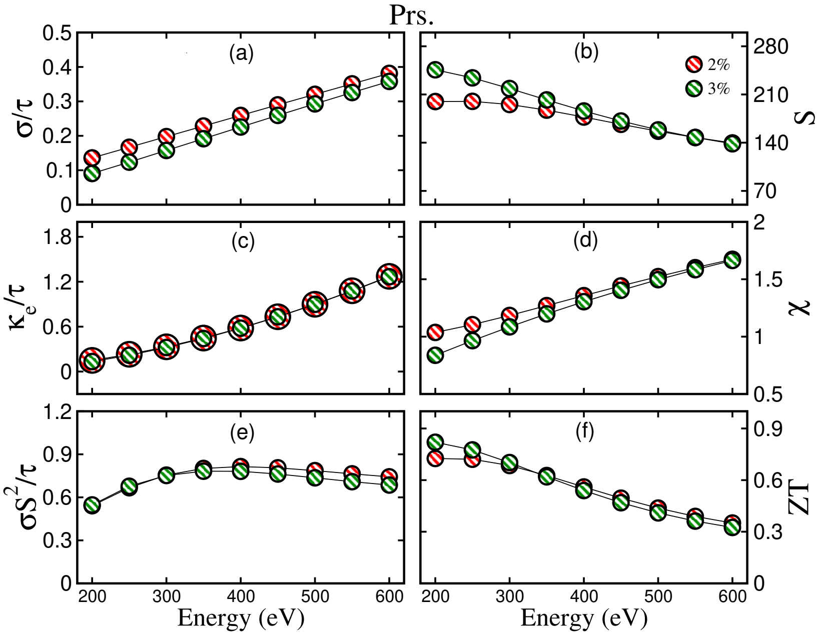

FIG. 11S: Computed (a) electrical conductivity per relaxation time ($\frac{\sigma}{\tau}$) in $\times 10^{19}$ 1/ms, (b) Seeback coefficient (S) in μV/K^{-1}, (c) thermal conductivity per relaxation time (κ_e/τ) in $\times 10^{14}$ W/mKs, (d) susceptibility (χ) in $\times 10^{-9}$ m^3/mol, (e) power factor (PF) in $\times 10^{11}$ W/mK^{2}s, and (f) figure of merit (ZT) in the prs. Sr_2CaOsO_6 double perovskite oxide under +2%/+3% biaxial ([110]) tensile strain.

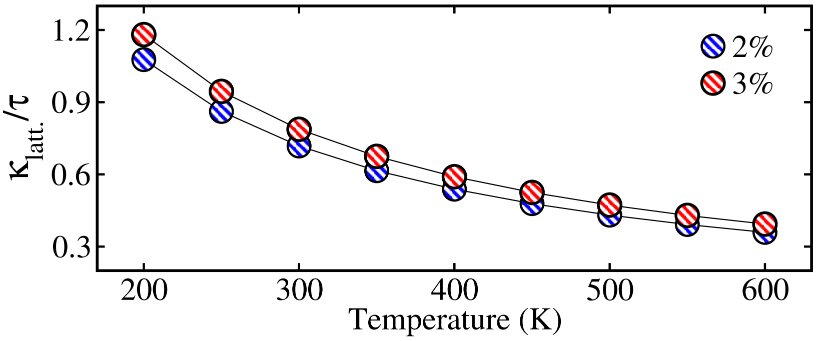

FIG. 12S: Computed value of lattice thermal conductivity per relaxation time ($\frac{\kappa_l}{\tau}$) in $\times 10^{14}$ Wm^{-1}K^{-1} under temperature in the prs. Sr_2CaOsO_6 structure.
